# Do medical students believe the back pain myths? A cross-sectional study

**DOI:** 10.1186/s12909-019-1676-x

**Published:** 2019-06-27

**Authors:** Eva McCabe, Dima Jadaan, Sudharshan Munigangaiah, Navya Basavaraju, John P. McCabe

**Affiliations:** 1 School of Medicine, National University of Ireland, Galway, Ireland; 2 Department of Trauma and Orthopaedic Surgery, University Hospital Galway, Galway, Ireland; 3 Department of Radiation Oncology, University Hospital Galway, Galway, Ireland; 4 Department of Trauma and Orthopaedic Surgery, University Hospital Galway, Galway, Ireland

## Abstract

**Background:**

Low back pain (LBP) is common, affecting 58–84% of adults at some point. In benign cases, misinformation can lead to harmful coping strategies and prolonged recovery time. Deyo has identified seven ‘Myths of Back Pain’ as misconceptions commonly seen in clinical practice of which doctors-in-training should be aware. We sought to determine medical students’ baseline knowledge of the prognosis and management of LBP compared to the general public and to dispel the ‘Myths of Back Pain’.

**Methods:**

We carried out a cross-sectional study of medical students (pre-clinical and clinical) at the National University of Ireland, Galway where students completed a questionnaire outlining the seven ‘Myths of Back Pain’. Final year students completed the survey before and after a seminar on LBP. Students’ results were compared with a random sample of the public who attended Galway University Hospital.

**Results:**

Two hundred nineteen students completed the questionnaire (59% female, 41% male). The mean age was 21 years (17–32). The mean number of correct answers increased according to medical school year (premedical 3/7, first year 4/7, final year 5/7). A personal history of back pain and female sex were associated with higher scores. On average, medical students answered 4/7 questions correctly overall, compared to the public (*n* = 131) who averaged at 3/7. Final years dispelled one further myth after their LBP seminar.

**Conclusions:**

Common misconceptions around LBP are prevalent among medical students and the general public. It is important that medical school curricula address these issues as part of their musculoskeletal programme.

## Background

Low back pain (LBP) is common and in fact, the most commonly reported site of pain [1]. It has been shown that 58–84% of adults will develop LBP during their lifetime [2]. Though the episodes may be severe, generally, they are short-lived and tend to resolve after 4–6 weeks [2]. LBP accounts for a significant proportion of medical clinic visits. It is the most common presenting symptom to primary care following upper respiratory symptoms [3]. While initially, the majority of cases should be managed conservatively at the primary care level, in practice, many patients are also treated for LBP in emergency departments. Patients are often advised to take time off work, rest, prescribed analgesics that may be inappropriate e.g. opioids and referred for premature imaging. Despite its prevalence, there have been no public health campaigns in Ireland to improve understanding of LBP.

It is accepted that negative attitudes and beliefs are important considerations in the development of LBP and disability [4, 5]. Misconceptions around its prognosis and management can reinforce negative coping mechanisms, prolong the recovery process and result in a considerable financial burden including medico-legal proceedings [6–8]. Based on these common misconceptions, Deyo introduced the seven ‘Myths of Back Pain’ [9]. These include the belief that it develops after injury (e.g. *“Most back pain is caused by injuries or heavy lifting”*), unrealistic expectations of diagnostic tests (e.g. *“Radiographs and newer imaging tests can always identify the cause of the pain”*) and unrealistic management expectations (e.g. *“If you have a slipped disc you must have surgery”*) [9].

Understanding health professionals’ attitudes towards LBP is important as they are associated with their patients’ beliefs, changing perceptions and adherence to evidence-based guidelines for investigation and management of back pain [10]. General practitioners in Ireland seek guidance from the National Institute for Health Care Excellence (NICE) in this regard given that there are no current Irish guidelines available [8, 11, 12]. However, there is only partial adherence to these recommendations [13]. Medical students typically learn about LBP by assimilation while they are rotating through general practice, rheumatology or orthopaedic surgery instead of receiving dedicated teaching on the subject. We sought to examine Irish medical students’ baseline knowledge of the prognosis and management of LBP compared to the general public and dispel the ‘Myths of Back Pain’.

## Methods

### Study design

This was a cross-sectional study carried out at the School of Medicine, National University of Ireland, Galway between January–April 2014. For a power of 80% and type I error rate of 5%, the required sample size to compare between medical students and the public was calculated as 61 medical students and 36 from the population if the sample size was unequal, or 46 of each group if the sample sizes were equal. To compare means of correct answers before and after teaching for final year medical students, the required sample size was calculated as 23 students.

### Study participants

The medical programme at NUI Galway is an undergraduate programme delivered over five/six years. All medical students receive standard training covering the musculoskeletal system beginning in first year with basic anatomy and physiology. Clinical teaching and rotations begin in year three. The dedicated musculoskeletal module including rheumatology and orthopaedics is delivered in the final year of the programme. Students may have encountered musculoskeletal complaints during their rotations in general practice, general internal medicine and emergency medicine up to this point.

The study participants were medical students from the pre-medical year, first and final year to compare the difference in understanding between pre-clinical and clinical year students. Final year students were completing the musculoskeletal module at the time of the study. All students gave verbal consent to participate in the study.

The control group was composed of a random sample of the public from previously published data by Munigangaiah et al., who attended Galway University Hospital, Ireland between April – August 2013 [14]. All members of the public were recruited randomly at point of entry to the hospital. All participants verbally consented to be included in the study.

### Study instrument

Deyo’s ‘Myths of Back Pain’ questionnaire includes the most common misconceptions encountered in clinical practice of which doctors-in-training should be aware [9]. It has been utilised in multiple studies as a succinct, quick assessment tool of attitudes towards low back pain [14–18]. The statements are described in Table 1. The responses were graded on a three point scale (agree, disagree, unsure). Given that all the statements were ‘myths’, participants received full marks if they disagreed with all seven statements. There are a variety of tools utilised to assess attitudes to back pain including the Pain Attitudes and Beliefs Scale and the Health Care Providers Pain and Impairment Relationship Scale. We used Deyo’s ‘Myths of Back Pain’ questionnaire because it had been previously used in the study of the public’s perceptions at our institution and would allow comparisons between these two populations.

**Table 1.** Deyo’s Seven Myths of Back Pain. Deyo, R.A. (1998) 'Low-back pain', Sci Am, 279(2), 48-53

1. If you have a slipped disc (also known as a herniated or ruptured disc), you must have surgery. 2. Radiographs and newer imaging tests (computed tomography [CT] and magnetic resonance imaging [MRI] scans) can always identify the cause of the pain. 3. If your back hurts, you should take it easy until the pain goes away. 4. Most back pain is caused by injuries or heavy lifting. 5. Back pain is usually disabling. 6. Everyone with back pain should have a spine radiograph. 7. Bed rest is the mainstay of therapy.	

### Procedures

Pre-medical and first year students were asked to complete the questionnaire during weekly lectures. The final year students completed the questionnaire before and at the end of a seminar on LBP during the musculoskeletal module delivered by the senior spine surgeon from the orthopaedic department. The seminar did not directly address the back pain myths but gave an overview of how to investigate and manage a patient with the condition. Participants completed the questionnaire anonymously. They were asked to provide their age, gender and whether they had a personal history of back pain. The researchers did not have access to identifiable information or influence over students’ examinations or grading.

### Ethical considerations

Ethical approval was granted for the study.

### Data analysis

Statistical analysis of the data was performed using R version 3.1.0 for Mac. Graphs were produced using Microsoft Excel version 14.4.4 for Mac. Members of the public and the medical students where matched by age and gender. Participants were excluded from the analysis if their questionnaires were not completed in their entirety. The differences in the frequencies of each answer for the seven myths were compared between age and gender groups using chi-squared tests. The differences in the mean number of correct answers between the medical school year using analysis of variance (ANOVA). The differences in the mean number of correct answers between males and females, between medical students and the general population and according to previous back pain experience were compared using student’s *t* tests. Comparisons between final year students before and after the seminar were made using independent sample tests and not paired tests as pairing was lost due to anonymity and differences in number of students (80 responded before talk, and 81 responded after talk). Multivariate regression analysis was used to examine the effect of medical school year and previous back pain experience with correct answers after controlling for age and gender. In all analyses, a two-sided *p* value less than 0.05 was considered statistically significant.

## Results

A total of 219/300 medical students completed the questionnaire in its entirety including 65 pre-medical, 74 first year and 80 final year students. The mean age of participants was 21 (17–32). 59% were female and 41% male. The mean number of questions answered correctly was 4.1 and 3.5 for females and males respectively, representing a statistically significant difference (*p* < 0.01, 95% CI: 0.16, 1.01).

The mean number of questions answered correctly increased according to medical school year including 3.25 for pre-medical students, 3.54 for first year medical students, and 4.66 for final year students (*p* < 0.0001). The final year students did significantly better to dispel the myths compared to their first year and pre-medical counterparts for all questions, except myth five (back pain is usually disabling).

132 (60%) students reported a personal history of back pain. The difference between the mean number of correct answers between those that had a personal history of back pain (4.05) and those that did not (3.6) was of borderline significance (*p* = 0.06, 95% CI: − 0.89, 0.02) after controlling for medical year, age and gender.

### Medical students’ responses versus the public

The results of the 219 medical students were compared to 131 members of the public who were matched for age and gender. The difference in mean between the correct answers given by the medical students (3.9) compared to the public (2.9) was statistically significant (*p* < 0.0001, 95% CI: 0.6, 1.4).

**Fig. 1.**
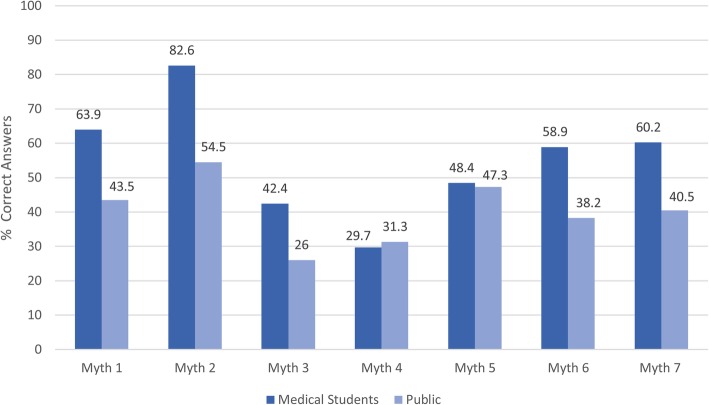
Number of Correct Answers: Medical Students vs. General Public

The differences in responses between medical students and the general population are shown in Fig. 1. The proportions of medical students who disagreed with the majority of the myths exceeded those of the public apart from myths four and five. For all myths, the differences in the frequencies of agreeing, disagreeing, and being unsure were significant (*p* < 0.05) except for myth number five, where responses were almost similar. Of note, almost 60% of the public and medical students agreed with myth four – most back pain is caused by injuries or heavy lifting.

### Difference in medical students’ perceptions of back pain after an educational session

**Fig. 2.**
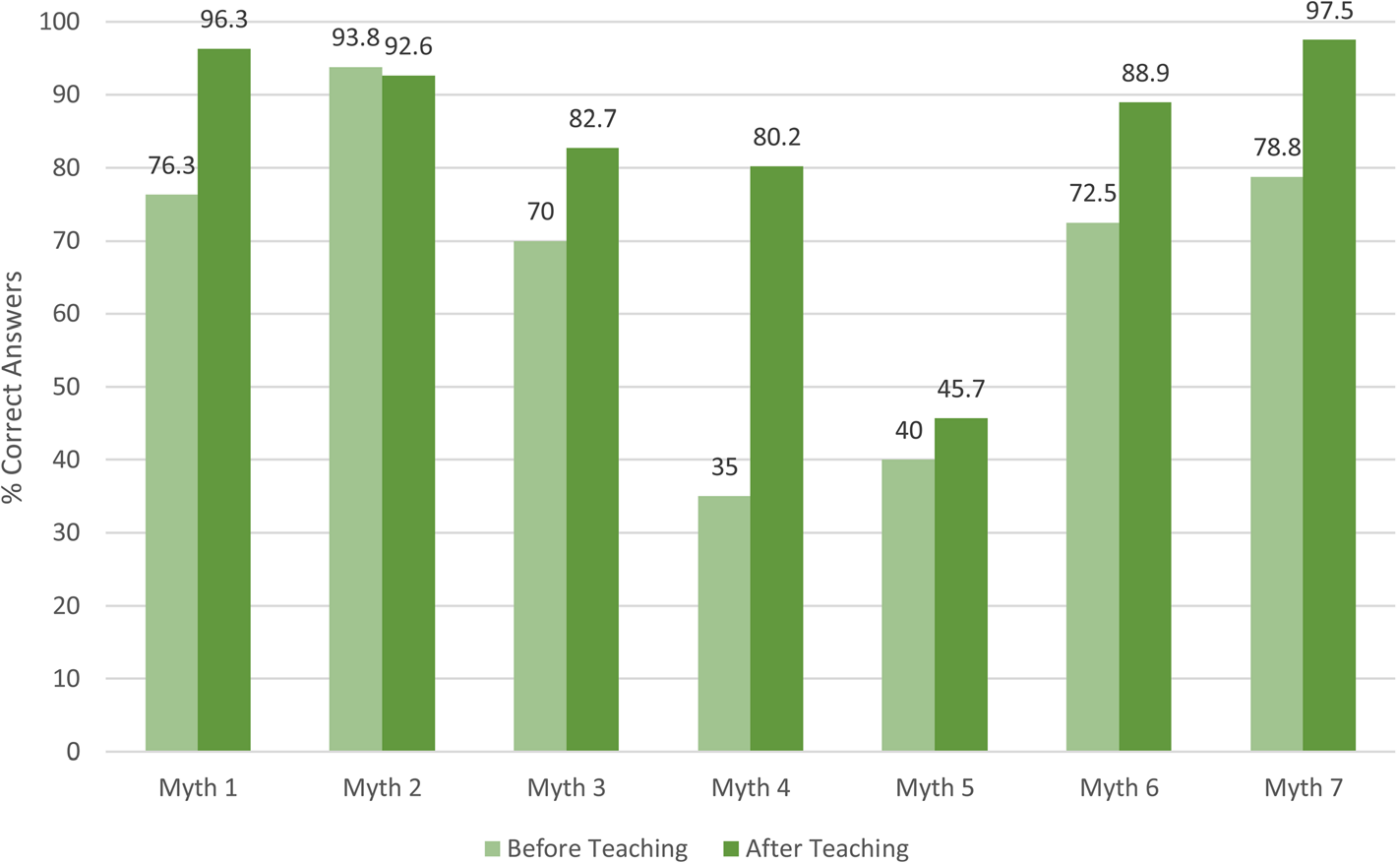
Number of Correct Answers: Before and After LBP Seminar

There was a small but significant improvement in final year results before (4.66) and after (5.84) the LBP seminar (*p* < 0.0001, 95% CI: 0.78, 1.58) as shown in Fig. 2. The largest improvement was for myth number four (most back pain is caused by injury or heavy lifting), where the students who disagreed with the statement after the seminar increased from 35 to 80%. For myth two (radiographs and newer imaging tests can always identify the cause of pain), a similar proportion of students surveyed before and after the seminar disagreed with the statement (93–94%).

## Discussion

Our results suggest that several back pain myths are believed to be true by medical students. “*Most back pain is caused by injuries or heavy lifting”* was the myth most commonly believed by our study cohort, however, it was largely dispelled following the LBP seminar. Factors that were associated with more correct responses included medical school year, female gender and a borderline association with personal history of back pain. The myth with the greatest difference in correct responses when comparing the medical students and the public was that *“Radiographs and newer imaging tests (computed tomography [CT] and magnetic resonance imaging [MRI] scans can always identify the cause of pain”.* Almost 60% of the general public believed that “*Bed rest is the mainstay of therapy”.*

These findings highlight a number of major misconceptions that exist among medical students and the public regarding the investigation and management of LBP. Myths that represent pain avoidance beliefs that may worsen the prognosis and lengthen the spontaneous recovery [7, 19, 20]. For example, bed rest is categorically known to prolong an acute episode of back pain [21]. Keeping active and returning to activities including work gradually is important in the recovery process. Believing that most LBP is caused by injuries or heavy lifting has a significant impact on work-related litigation rates. Unrealistic expectations on how LBP should be investigated by the public can harm to doctor-patient relationships. From a clinician standpoint, misconceptions may result in unnecessary radiological investigations. Spinal radiology does not necessarily determine the aetiology of pain in many situations and can expose patients to unnecessary radiation. Conversely, it may yield incidental findings that are of little significance, cause undue concern for clinicians and patients and result in increasing referrals to orthopaedics, rheumatology and neurosurgery.

Ihlebaek and Eriksen carried out a similar cross-sectional study in Norway where members of the public were sampled at random using telephone numbers and asked to complete Deyo’s questionnaire [15]. They found that those who had obtained a lower level of education had a different perception of LBP compared to those with who had higher education and answered more questions incorrectly. Only 12% of this cohort believed that “*bed rest is the mainstay of therapy*” versus 60% of the Irish public included in our study. The authors repeated their study after the introduction of national evidence-based guidelines and found a small improvement in survey responses [16]. Ihlehaeck and Eriksen also compared the public’s responses to those of general practitioners and physiotherapists after adjusting for level of educational attainment [17]. The health care professionals scored substantially higher but there was still significant uncertainty around use of imaging and necessity for surgery. Such uncertainty may continue to perpetuate misconceptions among the general public [17].

Our study did have a number of limitations. Firstly, we found that medical school year, female gender and personal experience of back pain were relevant factors in understanding perceptions towards LBP. However, this study was not designed to assess the impact of other individual student factors such as different backgrounds, life experience including pain experience, social learning, informal learning which may also be important to consider. Secondly, we did not carry out a long term follow-up to assess students’ delayed recall of LBP myths given that this may be variable after the learning stimulus. Thirdly, given the cross-sectional nature of this study, an approximation of understanding was obtained at one point in time. Finally, our results from students at NUI Galway may not be generalisable to other medical schools engaging in problem-based learning who may have more experience with dealing with LBP at an earlier stage in their training.

It is likely that most individuals will experience a musculoskeletal disorder at some point. Many of these conditions are chronic issues and may result in pain and disability, affecting their quality of life, activities and potentially interfering with their capacity to earn a living. Education in musculoskeletal medicine is necessary for all doctors including clinical assessment of common out-patient problems such as LBP. Limited exposure to these conditions may lead to students to interpret them as low priority. Further studies are needed to assess appropriate educational interventions to improve understanding and long term recall of LBP to ensure increased evidence-based approaches post-qualification.

## Conclusions

Widespread misconceptions exist regarding investigation and management of LBP among medical students and the public. Overall, we found that medical students were more successful at dispelling the ‘Myths of Back Pain’ compared to their lay counterparts. Year of medical training appeared to be associated with an increase in correct responses. Students received benefit from a targeted educational session on LBP. It is important that back pain myths are addressed in medical school curricula given the prevalence of the condition so that they do not persist and continue to permeate to the public.

## Data Availability

Data and materials are available from the corresponding author on request.

## Abbreviations

ANOVA: Analysis of variance
CT: Computed tomography
LBP: Low back pain
MRI: Magnetic Resonance Imaging
NICE: National Institute for Health Care Excellence
